# The First Record of Teredidae (Coleoptera, Coccinelloidea) from China, with Description of a New Species of *Teredus* Dejean, 1835 †

**DOI:** 10.3390/insects12111028

**Published:** 2021-11-15

**Authors:** Zhenhua Liu, Wei Lin, Zhiqiang Li

**Affiliations:** 1 Guangdong Key Laboratory of Animal Conservation and Resource Utilization, Guangdong Public Laboratory of Wild Animal Conservation and Utilization, Institute of Zoology, Guangdong Academy of Sciences, Guangzhou 510260, China; liuzhh_beetle@giz.gd.cn; 2 Technical Center of Gongbei Customs District, Zhuhai 519001, China; lw0525@163.com

**Keywords:** new record, East Asia, China, new species

## Abstract

**Simple Summary:**

As the largest group of organisms, Coleoptera includes more than 200 families worldwide; only 148 families have been recorded in China. Teredidae is a small family within Coccinelloidea, which has a cosmopolitan distribution, but has never been recorded in China. Until now, *Teredus* Dejean was endemic to Europe and North Africa. Here, we describe *Teredus chinensis* sp. nov. from central China, representing the first record of Teredidae in China and the first *Teredus* outside Europe and North Africa. This discovery not only enhances the Coleopteran diversity of China, but also largely extends the distribution of *Teredus*.

**Abstract:**

*Teredus* Dejean is a genus of the poorly known family Teredidae, which, historically, includes only two species, restricted to Europe and North Africa. *Teredus chinensis* sp. nov. is here described, representing the first member of Teredidae found in China, which significantly extends the distribution of *Teredus* to East Asia. The diagnostic characters and information about the wood boring beetles associated with the new species are provided.

## 1. Introduction

Teredidae is a small and poorly studied family of Coccinelloidea, consisting of 10 genera and about 160 extant species distributed worldwide—except for South America and Antarctica [1,2], with *Anommatus duodecimstriatus* (Müller) having been recently introduced to South America [3]. The adults and larvae of this family are commonly found under bark and in the galleries of wood boring beetles and were proposed to be mycophagous [4,5,6]; the members of Anommatinnae are usually collected from leaf litter or soil and their biology is unknown [1]. *Teredus* Dejean is a small genus of Teredidae which only includes two species, *T. cylindricus* (Olivier, 1790) and *T. opacus* Habelmann, 1854, restricted to Europe and North Africa. It can be easily distinguished from other genera within the family by the externally closed procoxal cavities.

*Teredus* was established by Dejean [7] to include *Lyctus nitidus* Fabricius, 1792 and *Ipis cylindrica* Oliver, 1790 in Colydiini of Colydiidae and *Ipis cylindrica* was recognized as the junior synonym of *Lyctus nitidus*. Ganglbauer [8] transferred *Teredus* to Derataphrini of Ceryloninae in Colydiidae and proposed the priority of *Teredus cylindricus*. *Teredus* remained in Colydiidae for a long time; Crowson [9] elevated Cerylonidae as a family in Clavicornia, while *Teredus* remained in Bothriderinae of Colydiidae. Though Bothrideridae was recognized as an independent family based on larval and adult characters [10,11], it was not widely accepted by other researchers. Lawrence [12] also discussed the family status of Bothrideridae and later formally proposed it [5], a proposition accepted by many researchers [1,6,13,14]. Teredidae was later split from Bothrideridae based on molecular data to accommodate the free-living taxa [2]. The monophyly of Teredidae was further supported by additional studies [15,16,17].

Recently, a series of *Teredus* specimens was collected from Shaanxi, China. These specimens can be easily distinguished from the known species; thus, a new species is here described.

## 2. Materials and Methods

Specimens were collected from blue and yellow pot traps, as well as from a ground trap, in Foping, Hanzhong, Shaanxi. The collected specimens were preserved in 75% ethanol. External structures and genitalic structures were examined by using a Leica Dvm6A stereomicroscope. Male genitalia, female abdomen and specimen for dissection were macerated in warm 10% KOH. Habitus images were taken using a Leica Dvm6A and edited in Photoshop (version CC2015). Individual structures in glycerol were photographed using a Zeiss AxioCam HRc digital camera mounted on a Zeiss AX10 microscope with the Axio Vision SE64 software and layers were then aligned in Helicon focus; scanning electron micrographs were taken with a Phenom Pro desktop scanning electron microscope, then aligned in Helicon Focus; aligned images were processed in Photoshop CC 2019.

The terminology used in this paper is here described [1,18]. Measurements were made as follows: body length—from the apical edge of the clypeus to the apex of the elytra; head width—width across the eyes; pronotal length—median line from the anterior margin to the posterior margin; pronotal width—maximum width of pronotum; elytral length—from the base of the scutellum to the elytral apex along the suture; elytral width– maximum width across the elytra.

Specimens involved in the study are being deposited in the following institutions:

FSCA—Florida State Collection of Arthropods, Gainesville, Florida, USA;
GDZI—Institute of Zoology, Guangdong Academy of Sciences, Guangzhou, China;
IZAS—Insitute of Zoology, Chinese Academy of Sciences, Beijing, China;
IOZ—Chinese National Zoological Museum of China, Academy of Sciences, Institute of Zoology, Beijing, China;
KIZ—Kunming Institute of Zoology. CAS, Kunming, China;
NHMUK—The Natural History Museum, London, UK;
SYSBM—The Museum of Biology, Sun Yat-sen University, Guangzhou, China;
ZIN—Russian Academy of Sciences, Zoological Institute, St. Petersburg, Russia.

## 3. Results

**Teredidae** Seidlitz, 1888.

***Teredus*** Dejean, 1835.

Type of species: *Lyctus cylindrica* Olivier, 1790.

**Diagnosis:** This genus can be distinguished from the other genera of Teredidae by the following characters: externally closed procoxal cavities; pronotum without basal impression and prosternum without anterior process; all tibiae without spines along outer edges.

*Teredus chinensis* sp. nov.

Type of Material Examined

**Holotype** (♂): 33°40′9.81″ N, 107°48′48.71″ E, Shaanxi Prov. Qinling Mts. Foping National Reserve, near Sanguan Temple, 1678 m; late June 2020; blue pot trap in *Bashania fargesii* forest Wangang Liu and Jianlong Chen leg. (1, SYSBM).

***Paratypes***: 33°40′9.81″ N, 107°48′48.71″ E, Shaanxi Prov. Qinling Mts. Foping National Reserve, near Sanguan Temple, 1678 m; late June 2020; blue pot trap in *Bashania fargesii* forest, Wangang Liu and Jianlong Chen leg. (1, SYSBM). At 33°40′11.66″ N, 107°48′46.12″ E, Shaanxi Prov. Qinling Mts. Foping National Reserve, near Sanguan Temple, 1698 m; late June 2020; yellow pot trap in *Bashania fargesii* forests, Wangang Liu and Jianlong Chen leg. (2; IOZ(E)225777–225778). At 33°40′58.66″ N, 107°51′0.49″ E, Shaanxi Prov. Qinling Mts. Foping National Reserve, Liangfeng Ya-Sanguan Temple, 2017 m; early July 2020; blue pot trap in *Fargesia qinlingensis* forests, Wangang Liu and Jianlong Chen leg. (1, FSCA). At 33°40′54.37″ N, 107°50′53.04″ E, Shaanxi Prov. Qinling Mts. Foping National Reserve, Liangfeng Ya-Sanguan Temple, 2008 m; early July 2020; blue pot trap in *Fargesia qinlingensis* forests, Wangang Liu and Jianlong Chen leg. (2; KIZ 0121457–0121458). At 33°40′50.48″ N, 107°50′44.42″ E, Shaanxi Prov. Qinling Mts. Foping National Reserve, Liangfeng Ya-Sanguan Temple, 1966 m; early July 2020; yellow pot trap in *Fargesia qinlingensis* forests, Wangang Liu and Jianlong Chen leg. (1; IZAS). At 33°38′50.55″ N, 107°48′22.76″ E, Shaanxi Prov. Qinling Mts. Foping National Reserve, near Sanguan Temple, 1580 m; late August 2020; yellow pot trap in *Bashania fargesii* forests, Wangang Liu and Jianlong Chen leg. (1; NHMUK). At 33°40′37.79″ N, 107°49′15.62″ E, Shaanxi Prov. Qinling Mts. Foping National Reserve, Liangfeng Ya-Sanguan Temple, 1777 m; early August 2020; yellow pot trap in *Fargesia qinlingensis* forests, Wangang Liu and Jianlong Chen leg. (1; ZIN). At Shaanxi Prov. Qinling Mts. Foping National Reserve, 1299–2017 m; June–September 2020; trap in bamboo forest, Wangang Liu and Jianlong Chen leg. (6, SYSBM; 15, GDZI).

**Etymology.** The species name refers to China, as this is the first species of *Teredus* and even the first described Teredidae from China.

**Type locality**. China, Shaanxi, Hanzhong, Foping, Foping National Reserve (33°40′9.81″ N, 107°48′48.71″ E).

**Figure 1:**
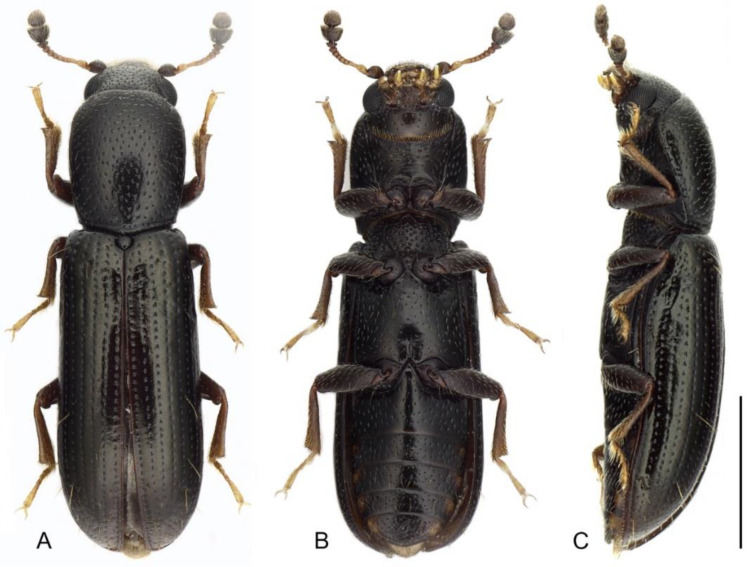
*Teredus chinensis* sp. nov., holotype, male: (**A**) dorsal view; (**B**) ventral view; (**C**) lateral view. Scale bar: 1 mm.

**Diagnosis.***Teredus chinensis* can be easily distinguished from the known species of *Teredus* by the existence of several pairs of long setae on the elytra (Figure 1A,C). In addition, the coarser punctations on the prontum and elytra, the dark brown legs and the bicoloured antennae are distinctly different from those of *T. cylindricus* and *T. opacus*.

Description

With a length of 3.0–4.4 mm, the body (Figure 1A,C) is distinctly cylindrical, parallel-sided; the colour is nearly uniformly black, except for the tarsi and antennomeres 2–8, which are brownish; the body shows a vestiture of short yellowish setae and longer bristles.

**Figure 2:**
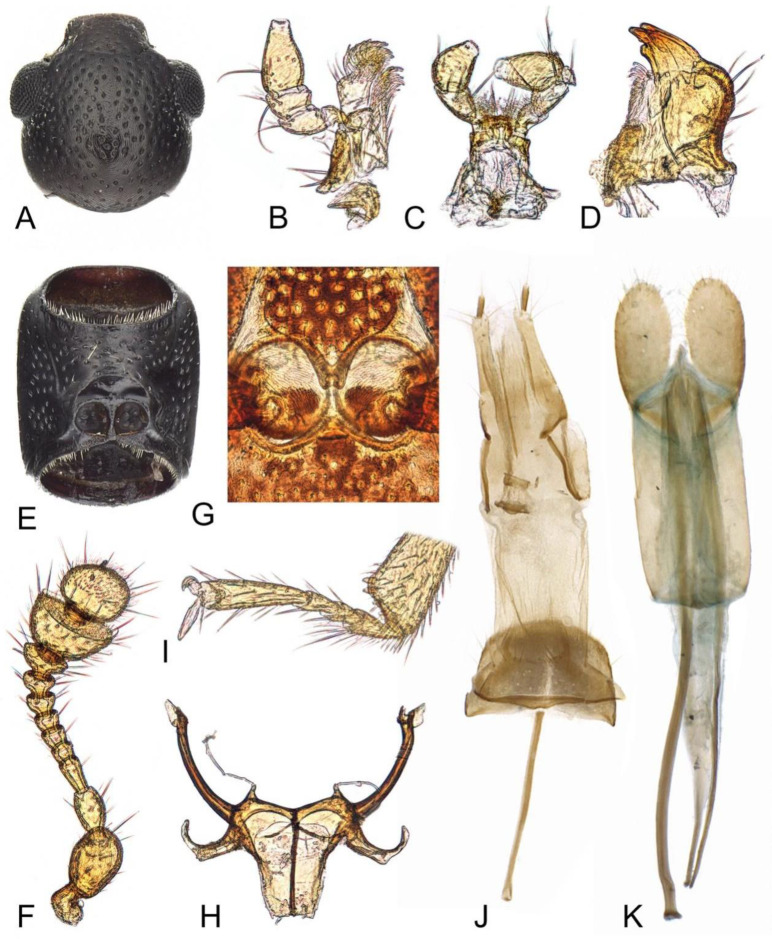
*Teredus chinensis* sp. nov., paratypes: (**A**) head, dorsal; (**B**) maxilla, ventral; (**C**) labium, ventral; (**D**) mandible, dorsal; (**E**) prothorax, ventral; (**F**) antenna; (**G**) meso–metaventral junction; (**H**) metendosternite, ventral; (**I**) hind tarsi; (**J**) female genitalia; (**K**) male genitalia.

**Figure 3:**
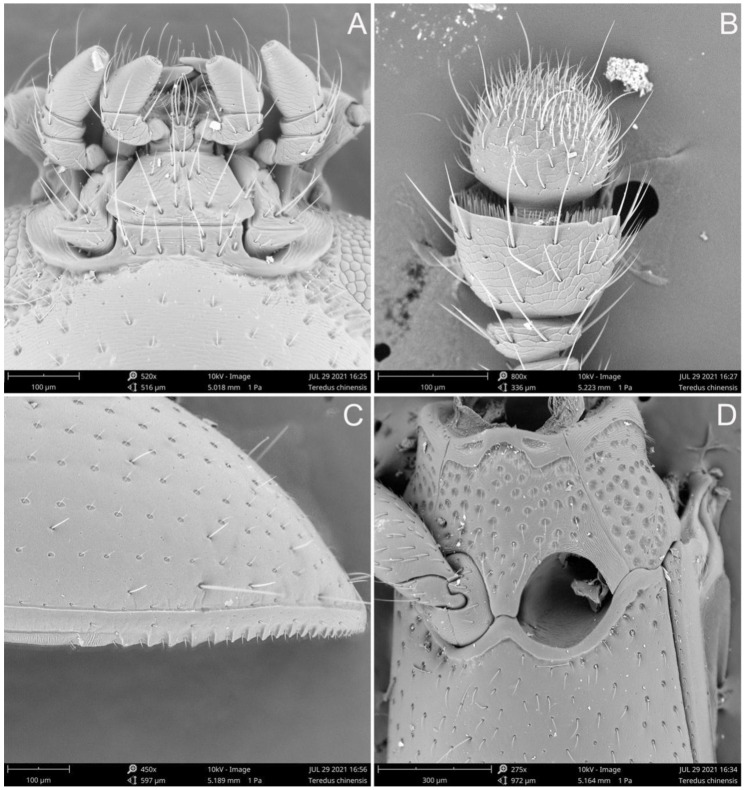
*Teredus chinensis* sp. nov., paratype, SEM images: (**A**) mouthpart, ventral; (**B**) antennal club; (**C**) apex of outer elytral margin; (**D**) mesoventrite and mesocoxae, ventral.

**Male.** The head (Figure 2A) is prognathous, widest across the eyes; it shows a pair of shallow and widely separated occipital incisions; a distinct frontoclypeal suture is absent, represented as impression. The eyes are finely facetted, slightly protruding. The antennae are laterally closely, inserted in front of the eyes. The antennae (Figure 2F) are 11-segmented, with a 2-segmentel club; the scape is enlarged, the pedicel is slightly enlarged anteriorly, antennomere 3 is nearly twice as long as antennomere 4 and, whereas antennomere 9 is strongly transverse, antennomere 10 is truncate and grooved apically, bearing dense sensorial setae (Figure 3B); the last antennomere is covered with dense short sensorial setae on the apical half. The clypeus is rectangular and transverse; the apex is nearly truncate. The mandible (Figure 2D) is short and stout, sub-triangular; the apex is tridentate; the prostheca is well developed, represented as tufted setae; the mola is also well developed. The maxilla (Figure 2B) has short 4-segmented palps; the last palpomere is sub-conical, the apex narrowed; the galea is distinctly broader than the lacinia, covered with dense setae anteriorly; the lacinia is slender, with the uncus, covered with dense setae anteriorly. The labium (Figure 2C) has 3-segmented palps; the first palpomere is short and small, while the second palpomere is expanded apically and the last palpomere is enlarged, with the apex slightly narrowed. The mentum (Figure 3A) is trapezoid.

The prothorax is slightly elongated, sub-rectangular and about 1.3 times as long as it is wide. The pronotum is slightly convex dorsally, covered with moderately dense and coarse punctations; the lateral carinae is complete. The notosternal suture is also complete. The prosternum in front of the procoxae is about 2.0 times as long as the procoxal cavities; the prosternal process is narrow and complete, the apex narrowly truncate. The procoxal cavities are rounded and narrowly separated, externally closed (Figure 2B); the procoxae is rounded and slightly projecting, while the protrochantins are completely concealed. The scutellum is small and sub-rounded.

The elytra are elongate and parallel-sided, about 2.3 times as long as they are wide, with two elytra combined, completely covering the abdomen; the apical fifth of the lateral margins is serrate (Figure 3C); the dorsal surfaces are glabrous, with 10 rows of seriate punctations on each side, covered with a pair of long yellow bristles on the interstriae, between two and three pairs of bristles on the humeri, between one and two pairs of bristles on the medial areas and more than seven pairs of shorter bristles on the apical areas, symmetrical or not; the bristles on interstriae 4 and 6 are longer than the others; the epipleuron extends to the apex. The mesoventrite and mesanepisternum are covered with dense and large punctations (Figure 3D). the mesoventrite presents with a pair of procoxal rests anteriorly, well separated; the mesanepisternum is large and elongated, while the mesepimeron is small and transverse. The mesocoxal cavities are rounded and are separated by 0.3 times the width of the mesocoxal cavities, laterally closed by the meeting of mesoventrite and metaventrite; the mesocoxae are rounded and slightly projecting, while the mesotrochantins are concealed. The mesometaventral junction is monocondylic (Figure 2G). The metaventrite is glabrous, without distinct punctuation; the discrimen is present and short. The metanepisternum is narrowly exposed from the ventrum and is elongated. The metendosternite (Figure 2H) presents with a broad basal strut, well-developed anterior arms and lateral laminae and widely separated anterior tendons. The metacoxae is slightly transverse, narrowly separated. The legs present with enlarged femora; the tibiae are expanded apically, with short spines along the apical edges; the tarsal formula 4-4-4, with the first tarsomere distinctly longer than the proceeding segment, presents with basal tarsomeres ventrally covered with dense, short setae (Figure 2I).

The abdomen (Figure 1B) presents with five freely articulated ventrites; ventrite 1 is distinctly longer than ventrite 2, while ventrites 2–5 are nearly equal in length. The intercoxal process on ventrite 1 is triangular, the apex acute. Male genitalia (Figure 2K) present with parameres that are large and elliptical, articulated with tegmen and well separated from each other; the penis is slightly sclerotized and elongated, with a pair of basal struts and one slender flagellum.

**Female.** Similar to male externally, in the female, sexual dimorphism is absent. Sternite 8 present with a long spiculum ventrale, articulated at base. The ovipositor (Figure 2J) is elongated and the gonocoxites are divided into slender distal gonocoxites and short proximal gonocoxites, bearing slender styli anteriorly; the paraprocts and baculus are moderately long.

Key to Species of Teredus Dejean, 1835.

1. The elytra present with several pairs of long setae, apical fifth of lateral margins serrate; the punctation on pronotum and elytra are relatively coarser···················*Teredus chinensis* sp. nov.
  - The elytra are glabrous, without long setae; the lateral margins are smooth to the apex; the punctuation on pronotum and elytra are fine································· ····························2.
2. The body presents with a rufous dorsum, similar to the colour of antennae and legs; the pronotum is slightly constricted near the base···················································*Teredus opacus* Habelmann.
  - The body presents with a nearly black dorsum, distinctly different from the brownish antennae and legs; the pronotum is not constricted near the base················*Teredus cylindricus* (Oliver).

## 4. Discussion

The loose antennal club and externally closed procoxal cavities can easily assign the new species to the genus *Teredus*, while the paired long setae on the elytra have never been recorded in this genus and exist in some species of *Teredolaemus* [19]. The presence of setae on the elytra is not regarded as a generic character as there are also some glabrous species in *Teredolaemus*. Furthermore, the serrate apical margin of the elytra is here described in Teredidae for the first time with function unknown, which makes *T. chinensis* also distinguishable from the other two congeners. Consequently, the description of this species also extends the definition of *Teredus*.

*Teredus chinensis* represents the first species of Teredidae from China, which is also the first record of *Teredus* outside of Europe and North Africa. The new species not only enhances the beetle diversity of China, but also largely expands the distribution of *Teredus*. Consequently, there is a large gap between the European and African species and the Chinese species, but more species are expected to be found in intermediate areas.

Members of *Teredus* and *Teredolaemus* have typically been collected in the tunnels of wood boring beetles such as Ptinidae (Anobiinae) and Curculionidae (Platypodinae and Scolytinae) [1,4,5]. Though the specimens in this study were collected by traps as mentioned above, a large number of wood boring beetles was found in the same traps, such as Scolytinae (*Xylosandrus germanus* (Blandford), *Xylosandrus amputates* (Blandford), *Xylosandrus borealis* Nobuchi, *Scolytoplatypus* cf. *blandfordi* Gebhardt, *Scolytoplatypus mikado* Blandford) and Bostrichidae (*Dinoderus japonicus* Lesné). Thus, *T. chinensis* probably has similar habitats as the other *Teredus* and *Teredolaemus*, feeding on the fungi in the galleries of some wood boring beetles.

## Data Availability

No new data were created or analysed in this study. Data sharing is not applicable to this article.
